# Luxation antéro-supérieure de l´épaule: à propos d´un cas et revue de la littérature

**DOI:** 10.11604/pamj.2021.40.7.26748

**Published:** 2021-09-02

**Authors:** Fahd Idarrha, Yassine Fath El Khir, Ahmed idoukitar, Mehdi Maskouf, Abdesslam Achkoun, Mohammed Amine Benhima, Imad Abkari, Halim Saidi

**Affiliations:** 1 Service de Traumatologie et d´Orthopédie, Hôpital Arrazi, Centre Hospitalier Universitaire Mohammed VI Marrakech, Marrakech, Maroc,; 2 Service de Traumatologie et d´Orthopédie à la Faculté de Médecine et de Pharmacie, Université Cadi Ayyad, Marrakech, Maroc

**Keywords:** luxation de l’épaule, luxation antéro-supérieure, luxation gléno-humérale, traumatisme de l’épaule, à propos d’un cas, Shoulder dislocation, anterior-superior dislocation, glenohumeral dislocation, shoulder trauma, a case report

## Abstract

Les luxations traumatiques de l'articulation de l'épaule sont généralement décrites comme des luxations antéro-inférieures. Les luxations antéro-supérieures sont extrêmement rares. Nous rapportons un cas rare de luxation traumatique de l´épaule antéro-supérieure chez un patient âgé de 45 ans où l´examen physique trouve une saillie antéro-supérieure de la tête humérale en sous-cutanée avec comblement de l´espace sous acromiale, le diagnostic était confirmé radiologiquement avec la découverte d´une rupture totale des tendons supra-épineux et sous scapulaire à l´imagerie par résonance magnétique. L´épaule était jugée instable après réduction de la luxation. Une arthrorise gléno-humérale provisoire était réalisée par la suite avec des bons résultats fonctionnels.

## Abstract (en)

Traumatic dislocations of the shoulder joint are generally described as an antero-inferior dislocations. Anterior-superior dislocations are extremely rare. We report a rare case of traumatic dislocation of the anterior-superior shoulder in a 45-year-old patient whose physical examination showed an anterior-superior subcutaneous protrusion of the head filling the subacromial space. The diagnosis was radiologically confirmed. MRI showed total rupture of the supraspinatus and subscapularis tendons. Shoulder instability occurred after reduction of the dislocation. Temporary glenohumeral arthrodesis was subsequently performed with good functional results.

## Introduction

Les luxations GH représentent environ 50% de toutes les luxations articulaires, 95% à 97% d'entre elles étant des luxations antérieures [1]. Les luxations antéro-supérieures sont extrêmement rares, car l'arc coracoacromial empêche anatomiquement la tête humérale de se déplacer crânialement. Nous rapportons un cas rare de luxation traumatique de l´épaule antéro-supérieure chez un patient âgé de 45 ans avec discussions de ses manifestations cliniques et la prise en charge chirurgicale.

## Patient et observation

**Présentation du patient**: nous rapportons le cas d´un patient âgé de 45 ans, sans antécédents pathologiques particuliers, qui se présente aux urgences suite à un accident de voie publique par moto, occasionnant chez lui un traumatisme de l´épaule droit, avec des brûlures du membre supérieur et inférieur droit et du tronc.

**Figure 1:**
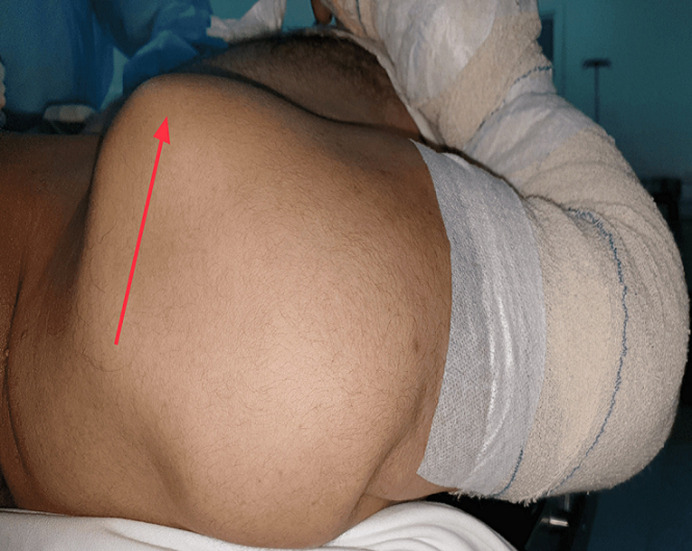
aspect clinique de la luxation antéro-supérieure de l´épaule, (flèche rouge: déplacement antéro-supérieure de la tête humérale)

**Figure 2:**
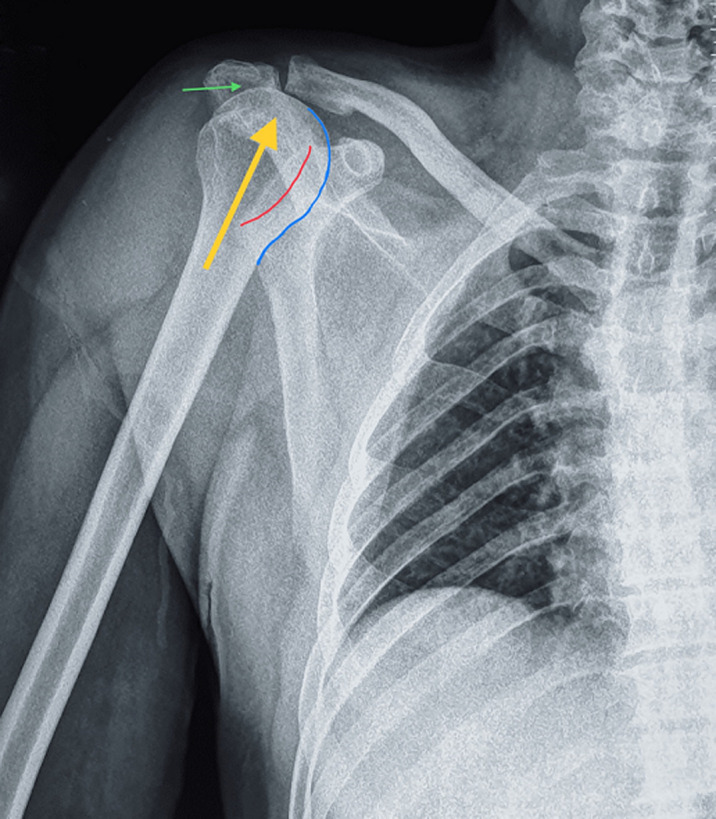
radiographie de face objectivant la luxation antéro-supérieure de l´épaule, (flèche jaune: le déplacement supérieur de la tête humérale; ligne rouge: la limite antérieure de la cavité glénoïde; ligne bleue: contour interne de la tête humérale; flèche verte: comblement de l´espace sous-acromiale par la tête humérale)

**Démarche diagnostique**: à l'examen physique, une saillie sous-cutanée antéro-supérieure de la tête humérale a été observée avec comblement de l´espace sous acromial (Figure 1), le signe de l'épaulette était présent correspondant à une modification du galbe de l´épaule en rapport avec la vacuité de la glène humérale. L'amplitude des mouvements de l'épaule n'a pas pu être examinée en raison de l´intensité de la douleur, l´examen vasculaire et nerveux en aval était sans particularités. La radiographie standard de l´épaule a confirmé la luxation antéro-supérieure de la tête humérale (Figure 2). L´imagerie par résonance magnétique (IRM) a révélé une rupture totale et transfixiante des tendons sus épineux et subscapulaire avec une interposition de la longue portion de biceps.

**Figure 3:**
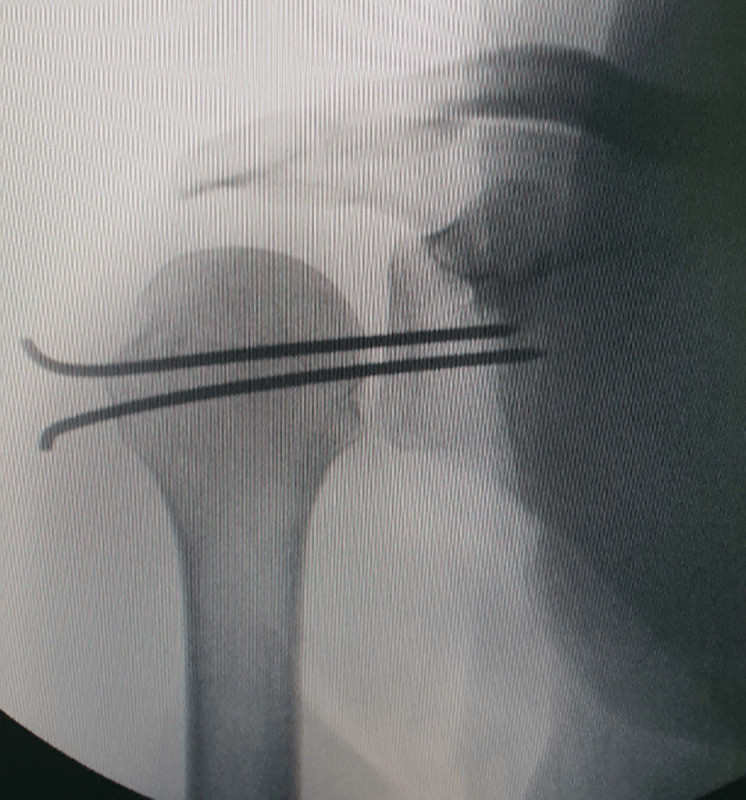
radiographie de face de l´épaule après réduction avec arthrorise gléno-humérale par 2 broches de Kirchner

**Intervention thérapeutique**: la réduction manuelle était facilement réalisée par une traction axiale caudale du bras avec compression antéro-postérieure de la tête humérale, cependant la tête humérale était extrêmement instable et se reluxe spontanément. Une arthrodèse provisoire de l'articulation de l'épaule était réalisée par la suite, sous anesthésie générale, la tête humérale a été stabilisée dans sa position anatomique par deux broches de Kirchner de taille 25/10° par voie percutanée (Figure 3).

**Suivi et résultats**: les broches de Kirchner étaient retirées après 6 semaines avec un testing de stabilité satisfaisant. Aucune récidive clinico-radiologique de luxation de l'épaule droite n'a été observée sur un recule de 6 mois, avec un score de DASH-Membre supérieur (Disabilities of the Arm, Shoulder and Hand) [2] évalué à 15,83 et 40 selon le score d´oxford d´instabilité de l´épaule (OSIS) [3].

**Consentement éclairé**: le patient a été informé sur cet article, sur la nature de l´étude, son but, sa durée et de la raison pour laquelle son cas était particulier. Il a volontairement donné son consentement éclairé pour permettre aux auteurs d'utiliser ses photos pour ce rapport de cas.

## Discussion

Les luxations supérieures de l'articulation gléno-humérale sont extrêmement rares. Très peu d'études ont été publiées sur la luxation supérieure de l'épaule. Stimson [4] a rapporté 14 cas dans la littérature avant 1912. La cause habituelle d'une luxation supérieure est une force axiale caudo-crâniale à direction antérieure sur un bras en adduction. Les radiographies révèlent un déplacement de la tête humérale au-dessus de la cavité glénoïde, souvent accompagné d'une fracture de la clavicule, de l'acromion, du coracoïde ou de la tubérosité supérieure de l'humérus, ou encore la séparation de l'articulation acromio-claviculaire.

La subluxation antéro-supérieure de l'articulation de l'épaule a été rapportée comme une complication de l'échec des opérations de la coiffe des rotateurs avec une combinaison d'insuffisance de la coiffe des rotateurs comprenant la rupture du subscapularis, la perte de l'arc coraco-acromial et la force de contractilité du deltoïde antérieur. Ogawa *et al*. [5] ont rapporté deux cas de subluxation antéro-supérieure causée par une contracture du deltoïde et la formation d'une bande fibreuse dans la partie postérieure du deltoïde convertissant le poids du bras en une force qui entraîne une luxation de la tête humérale dans la direction antéro-supérieure. Galatz *et al*. [6] ont rapporté une luxation antéro-supérieure postopératoire dans le cas d'une insuffisance massive de la coiffe des rotateurs. Matsuzaki *et al*. [7] ont décrit le cas d´une luxation antéro-supérieure de l´épaule post traumatique chez une patiente suivie pour la maladie de parkinson, cette luxation est expliquée par la rupture du subscapulaire et du supra-supinateurs.

La stabilité antéro-supérieure de l'épaule provient essentiellement des contraintes, primaires et secondaires empêchant la migration supérieure de la tête humérale. La principale contrainte, la coiffe des rotateurs, composée essentiellement du sous-scapulaire et du supra-supinateurs, cette migration supérieure de la tête humérale est corrélée à la taille et la localisation de la rupture de la coiffe des rotateurs [8]. L'arc coraco-acromial agit comme une contrainte secondaire à la migration supérieure, son rôle augmentant dans une épaule déficiente de coiffe des rotateurs [9]. La perte de la contrainte est compliquée par la conciliation de la partie antérieure du muscle deltoïde. La faiblesse contractile du deltoïde antérieur résulte d'un amincissement, d'une déhiscence ou d'une dénervation. Les parties postérieures et latérales intactes du deltoïde exercent un vecteur postérieur sur la tige médiane de l'humérus pendant la contraction, intensifiant la luxation antérieure de la tête humérale [10].

La subluxation antéro-supérieure peut également être exacerbée par une contracture capsulaire postérieure asymétrique. Pris ensemble, la perte de la coiffe des rotateurs, de l'arc coraco-acromial et du deltoïde antérieur entraîne une instabilité dynamique antéro-supérieure [10]. Dans le cas présent, la cause de la luxation antéro-supérieure peut être expliquée par la rupture des muscles de la coiffe des rotateurs post traumatiques, le sus-épineux et sous-scapulaire, qui sont la musculature de soutien associée à la stabilité de l'articulation de l'épaule. Chose qui peut être aggravée par les tentatives d´extirper le patient par son entourage du dessous de sa moto qui a pris feu lors de l´incident. L´approche chirurgicale adoptée est liée à l´état général du patient présentant des brûlures multiples et surinfectées ce qui rend l´exploration chirurgicale à ciel ouverte à haut risque de complication infectieuse. L´évolution après 6 mois du geste et les résultats des scores de DASH et OSIS supportent notre démarche thérapeutique initiale.

## Conclusion

Nous présentons dans ce travail le cas d'un patient qui présente une luxation antéro-supérieure de l´épaule qui est une forme clinique rare de luxation gléno-humérale, très peu décrite dans la littérature. C´est une luxation souvent accompagnée d'une fracture de l´arc acromio-claviculaire ou une rupture des tendons de la coiffe des rotateurs, sa prise en charge dépend du bilan lésionnel initial et de la stabilité de l´épaule après réduction.
